# Comparing levocardia and dextrocardia in fetuses with heterotaxy syndrome: prenatal features, clinical significance and outcomes

**DOI:** 10.1186/s12884-017-1579-y

**Published:** 2017-11-23

**Authors:** Xiaofang Wang, Yifan Shi, Shi Zeng, Jiawei Zhou, Jia Zhou, Hongxia Yuan, Lin Wang, Weiyuan Shi, Qichang Zhou

**Affiliations:** 1 Department of Ultrasound Diagnosis, The Second Xiangya Hospital, Central South University, 139 Renmin Road (M), Changsha, Hunan 410011 China; 2 Department of Ultrasonography, Chenzhou No.1 People’s hospital, Chenzhou, Hunan 423000 China; 3 Department of Ultrasonography, The first affiliated hospital of University of South China, Hengyang, Hunan 421001 China; 4 Department of Ultrasonography, Maternal & Child Health Center of Changsha, Changsha, Hunan 410011 China; 5 Department of Ultrasonography, Maternal & Child Health Center of Zhuzhou, Zhuzhou, Hunan 412000 China

**Keywords:** Heterotaxy, Levocardia, Dextrocardia, Prenatal diagnosis

## Abstract

**Background:**

To investigate the differences in cardiovascular disease, extracardiac anomalies and outcomes between fetuses with levocardia and dextrocardia.

**Methods:**

Clinical demographics, prenatal features, postnatal characteristics and the outcomes of fetuses with levocardia or dextrocardia were recorded and analyzed.

**Results:**

Sixty-five fetuses with dextrocardia and thirty-eight fetuses with levocardia were enrolled. Right ventricle outlet obstruction, atrioventricular septal defect and intestinal malrotation were common in both groups. Univentricular physiology, transposition of the great arteries and esophageal atresia were more frequent in fetuses with levocardia, whereas abnormal pulmonary venous connection, double outlet of right ventricle, left ventricle outlet obstruction and brain abnormalities were more frequent in the dextrocardia group. The accuracy of evaluating cardiac malformations was high, but the sensitivity in assessing extracardiac abnormalities was low.

**Conclusions:**

Although the disorders have certain overlapping features, there are several differences between fetuses with levocardia and dextrocardia. These findings might improve patient counseling and perinatal management.

## Background

Heterotaxy, also known as situs ambiguus, is the term used to describe the abnormality in left-right laterality in overall whole-body patterning. Heterotaxy differs from both normal visceral arrangement (situs solitus) and the mirror-image of organ anatomy (situs inversus). Heterotaxy syndrome, which occurs in 1 in every 6000 to 1 in every 20,000 live births [1], is a heterogeneous disorder associated with a wide spectrum of cardiac defects and extracardiac anomalies [2, 3]. Conventionally, the primary heterotaxy syndrome landmark is spleen abnormality because the spleen is almost always affected in patients. Currently, patients with heterotaxy syndrome are subdivided into “right isomerism” or “left isomerism” according to the characteristic morphology of the atrial appendages of the heart, bilateral symmetric lung lobes and bronchi [4]. Right isomerism is usually associated with complex cardiac defects, intestinal malrotation and absence of the spleen; left isomerism generally presents with an interrupted inferior vena cava, heart block and multiple spleens.

Although heterotaxy syndrome can be detected prenatally, it is difficult to classify these patients as “right isomerism” or “left isomerism” in utero. Atrial appendage morphology, lung lobation, bronchial branching and spleen abnormalities often escape prenatal detection. Considerable differences in cardiovascular disease, extracardiac anomalies, management procedure and postnatal outcome exist between the two groups [2, 3, 5, 6], leading to challenges when obstetric physicians consult affected patients.

However, the intrathoracic position of the heart can be detected easily and correctly prenatally as left sided, right sided or midline. Gindes L et al. [7] demonstrated fetal isolated levocardia is associated with a good outcome from three case series report, While Bohun CM et al. [10] reported common cardiac and non cardiac malformations in 81 cases with dextrocardia. Therefore, we hypotheses that there may be discrepancies in associated cardiac/noncardiac malformations and post-natal outcome between fetuses with levocardia and dextrocardia.

In this paper, we subdivided fetuses with heterotaxy into “levocardia” and “dextrocardia” groups according to the position of the heart, which is easily detected in utero, and attempted to compare the prenatal features, postnatal clinical characteristics and outcomes between the two groups to provide useful information for prenatal consultation.

## Methods

A retrospective, multicenter follow-up investigation was conducted at The Second Xiangya Hospital of Central South University in China, the Chenzhou No.1 People’s Hospital in China, and the First Affiliated Hospital of University of South China, Maternal & child health center of Changsha and Zhuzhou from January 2000 to June 2016. Fetuses with heterotaxy syndrome were included in this study and were identified by an abnormal cardiovisceral left-to-right axis arrangement. The exclusion criteria were fetuses with situs solitus and fetuses with situs inversus. Baseline demographics, prenatal findings, postnatal diagnosis, management and outcomes were recorded and analyzed. Written informed consent was obtained from all of the families. The study was approved by the ethics committee of the fourth hospitals.

Comprehensive examination was performed for each fetus with suspected heterotaxy syndrome using the following standard protocols: (1) identification of left-right laterality of fetal position; (2) position of the thoracic and abdominal organs, including heart, heart apex, stomach, liver, gallbladder, portal sinus, descending aorta and inferior vena cava; (3) position and number of spleen, if present; (4) morphology of atrial appendage, if present; (5) segmental approach for the detection of cardiac structural and rhythmic abnormality; (6) presence of extracardiac anomaly.

**Fig. 1:**
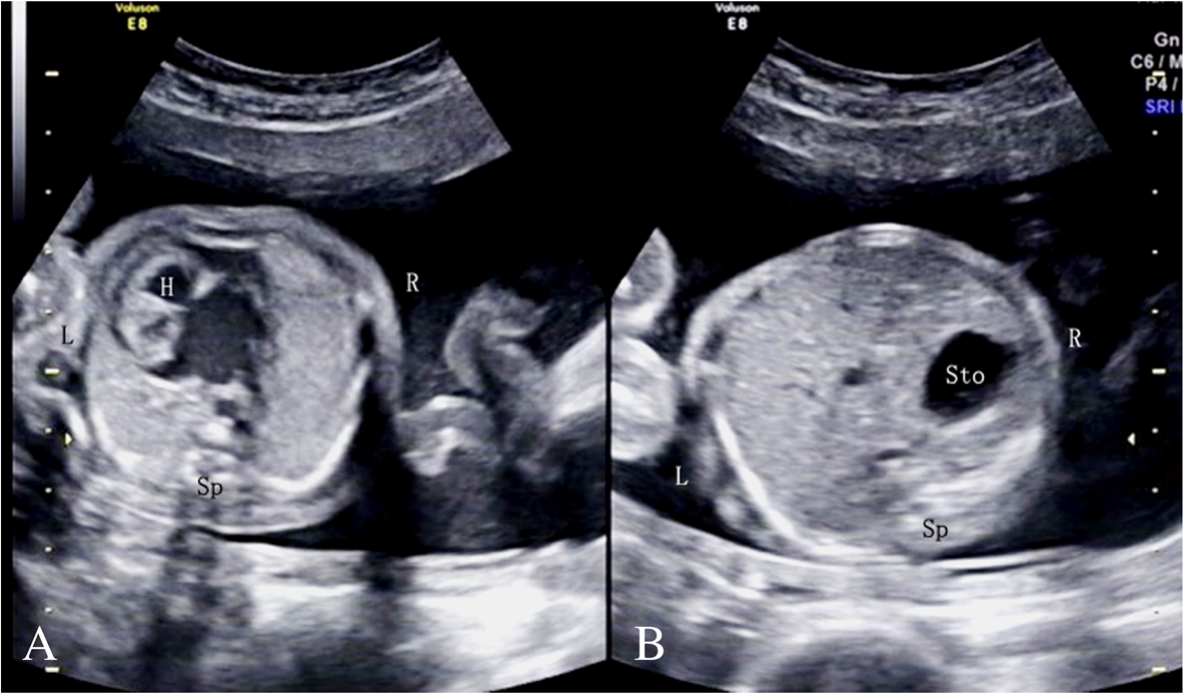
US show a fetus at the gestational age of 21.1 weeks with levocardia. The heart is located in the normal levo position with a left-sided cardiac apex (shown on **a**). The stomach is located in the right side of the abdomen (shown in **b**)

**Fig. 2:**
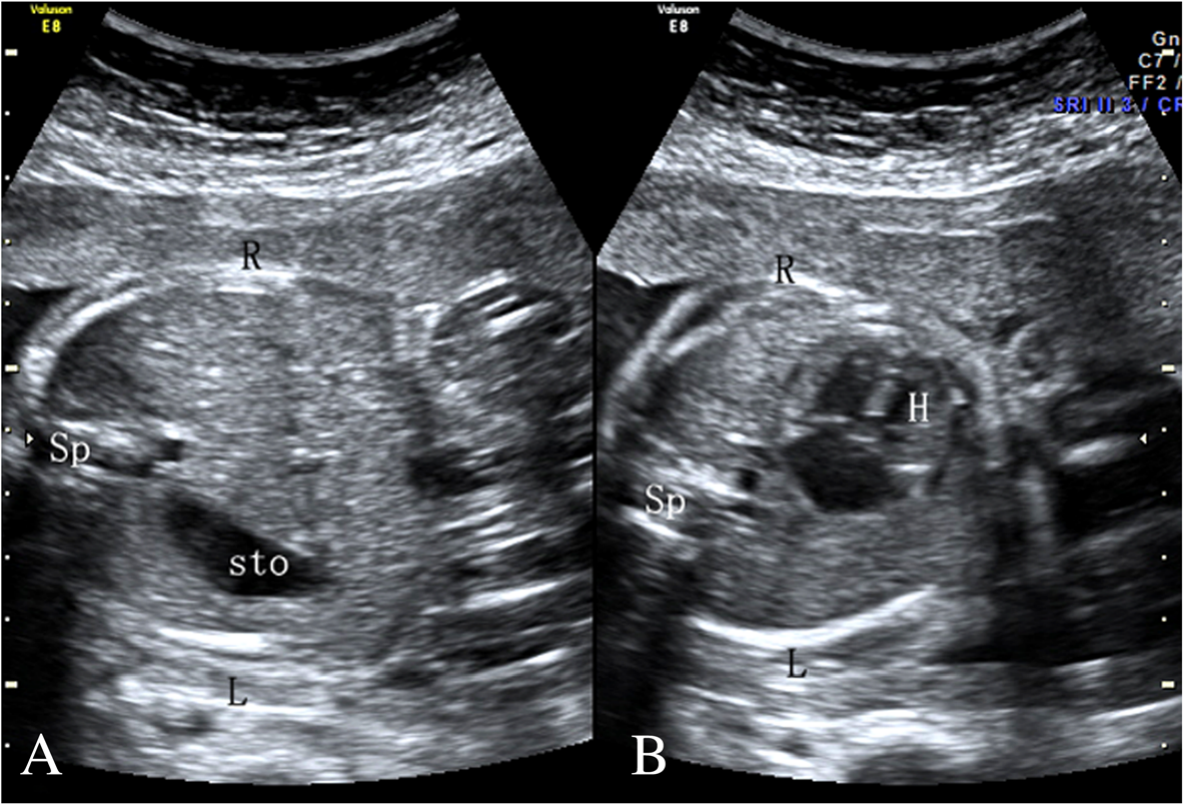
US shows a fetus at the gestational age of 22.7 weeks with dextrocardia. The heart is located in the right hemithorax with the heart axis directed to the right and caudad (shown on **a**). The stomach is located in the left side of the abdomen (shown in **b**)

When a fetus presented with situs ambiguus (discordant laterality of the heart, stomach, portal sinus, or gallbladder), prenatal heterotaxy was diagnosed. For the purpose of this study, fetuses with heterotaxy were subdivided into levocardia (normal levo position with left-sided cardiac apex [7], Fig. 1) and dextrocardia (located in the right hemithorax with heart axis directed to the right and caudad [8], Fig. 2) groups. Karyotype examination was advised, and the parents were counseled on the option of pregnancy termination or continuation. The autopsy findings were recorded in cases of termination. At the end of the study period, all of the identified patients received a standardized telephone call follow-up with particular emphasis on postnatal findings (e.g., electrocardiogram, echocardiography, CT, MRI), management and current medications.

The data are presented as the means ± SDs or frequencies (percent). The prevalence of heterotaxy syndrome is reported as the percentage of all included patients. Comparisons for continuous variables were performed using unpaired, two-tailed Student’s t-tests and χ^2^ tests or Fisher’s exact test for categorical variables. A probability value of *P* < 0.05 was considered statistically significant.

## Result

Between 1 January 2000 and 30 June 2016, 682,177 consecutive pregnant women with a total of 714,306 fetuses were referred for echocardiography at the three institutions. A total of 103 fetuses with heterotaxy syndrome were identified and enrolled in our study, resulting in a prevalence of 0.014%. Sixty-five fetuses exhibited dextrocardia, and thirty-eight fetuses had levocardia. The clinical characteristics and prenatal features are summarized in Table 1.

**Table 1:** Prenatal and perinatal outcome of fetuses with levocardia and dextrocardia

Characteristics	Fetus with levocardia (*n* = 38)	Fetus with dextrocardia (*n* = 65)	*P*
Baseline demographics			
Maternal age, y	26.7 ± 5.2	27.1 ± 4.5	0.69
Nulliparity, n (%)	24 (63.1%)	51 (78.5%)	0.11
Singleton, n (%)	35 (92.1%)	61 (89.7%)	0.71
ART, n (%)	6 (15.8%)	10 (15.4%)	1.0
Family history of CHD, n (%)	8 (21.1%)	12 (18.5%)	0.79
Diabetes, n (%)	15 (39.5%)	24 (36.9%)	0.84
Gestational age at diagnosis, wk	22.4 ± 1.5	22.6 ± 1.3	0.32
EFW, g	540.5 ± 135.7	555.0 ± 105.0	0.55
Sex: Males, n (%)	29 (76.3%)	42 (64.6%)	0.27
Gestational age at birth, wk	38.3 ± 2.6	39.5 ± 2.3	0.64
Birth weight, g	2817.2 ± 246.3	2789.4 ± 209.3	0.59
Prenatal features			
Abnormal situs			
Left stomach, n (%)	0	15 (23.1%)	*P* < 0.01
Right stomach, n (%)	38 (100%)	50 (76.9%)	*P* < 0.01
Midline liver, n (%)	24 (63.2%)	39 (60%)	0.83
Juxtaposition of aorta and IVC, n (%)	30 (78.9%)	32 (49.2%)	*P* < 0.01
Cardiovascular issue			
Without defect, n (%)	2 (5.3%)	0	0.13
Any defect, n (%)	36 (94.7%)	65 (100%)	0.13
Interrupted IVC, n (%)	7 (18.4%)	18 (27.7%)	0.35
Abnormal SVC, n (%)	9 (23.7%)	36 (55.4%)	*P* < 0.01
APVC, n (%)	9 (23.7%)	35 (53.8%)	*P* < 0.01
AVSD, n (%)	20 (52.6%)	40 (61.5%)	0.41
Common atrium, n (%)	15 (39.5%)	24 (36.9%)	0.83
Univentricular physiology, n (%)	25 (65.8%)	16 (24.6%)	*P* < 0.01
TGA, n (%)	14 (36.8%)	4 (6.1%)	*P* < 0.01
DORV, n (%)	4 (10.5%)	37 (56.9%)	*P* < 0.01
DOLV, n (%)	0	1 (1.5%)	1.0
Truncus arteriosus, n (%)	2 (5.3%)	0	0.13
RV outlet obstruction, n (%)	25 (65.8%)	41 (63.1%)	0.83
LV outlet obstruction, n (%)	1 (2.6%)	11 (16.9%)	*P* < 0.05
Aortic arch anomaly, n (%)	6 (15.8%)	6 (9.2%)	0.35
Arrhythmias, n (%)	4 (10.5%)	17 (26.1%)	0.08
Extracardiac issue			
Without malformation, n (%)	35 (92.1%)	59 (90.8%)	1.0
Any malformation, n (%)	3 (7.9%)	6 (9.2%)	1.0
Esophageal atresia, n (%)	1 (2.6%)	0	0.37
Intestinal atresia, n (%)	0	1 (1.5%)	1.0
Duodenal atresia, n (%)	0	1 (1.5%)	1.0
Hernia, n (%)	0	1 (1.5%)	1.0
Renal lesion, n (%)	1 (2.6%)	1 (1.5%)	1.0
Single UA, n (%)	2 (5.3%)	3 (4.6%)	1.0
Bone malformation, n (%)	0	2 (3.1%)	0.53
Hydrops, n (%)	3 (7.9%)	9 (13.8%)	0.53
Chromosomal anomalies, n (%)	4 (10.5%)	5 (7.7%)	0.72
Perinatal outcome			
IUFD, n (%)	7 (18.4%)	12 (18.5%)	1.0
TOP, n (%)	11 (28.9%)	24 (36.9%)	0.52
Live birth, n (%)	20 (52.7%)	29 (44.6%)	0.54

*ART* assisted reproductive technique, *APVC* abnormal pulmonary venous connection, *AVSD* atrioventricular septal defect, *CHD* congenital heart disease, *DORV* double outlet of right ventricle, *DOLV* double outlet of left ventricle, *EFW* estimated fetal weight, *IUFD* intrauterine fetal death, *IVC* inferior vena cava, *SVC* superior vena cava, *TOP* termination of pregnancy, *TGA* transposition of the great arteries, *NND* neonatal death

There were no differences in maternal age, pregnancy history, and fetal sex between the two groups. There was a high rate of diabetes and family history of congenital heart defects both in fetuses with levocardia and fetuses with dextrocardia (39.5%, 36.9% for diabetes and 21.1%, 18.5% for family history of CHD, respectively).

All of the fetuses with levocardia presented with a right-sided stomach, and most fetuses with dextrocardia (76.9%) presented with a right-sided stomach. There were no differences in liver position or juxtaposition of the aorta and inferior vena cava between the two groups.

Cardiovascular deformations were present prenatally in all of the enrolled fetuses except for two with levocardia. There were more fetuses with univentricular physiology and transposition of the great arteries in the levocardia group compared with the dextrocardia group (*P* < 0.01). Furthermore, more fetuses in the dextrocardia group had double outlet of right ventricle, left ventricle outlet obstruction and abnormal pulmonary venous connection compared with the levocardia group (P < 0.01). In the group with levocardia, the types of CHD were distributed as follows: univentricular physiology (25/38, 65.8%); right ventricle outlet obstruction (25/38, 65.8%); atrioventricular septal defect (20/38, 52.6%); common atrium (15/38, 39.5%); TGA (9/38, 36.8%); APVC (9/38, 23.7%); and abnormal superior vena cava (9/38, 23.7%). Univentricular physiology included 9 cases with tricuspid atresia, 6 cases with bicuspid atresia and 10 cases with an unbalanced common atrioventricular valve. RV outlet obstruction included 11 cases with pulmonary valve stenosis and 15 cases with pulmonary artery atresia. APVC included 7 cases with total anomalous pulmonary venous connection and 2 cases with partial anomalous pulmonary venous connection. Abnormal superior vena cava included 6 cases with double SVC and 3 cases with persistent left SVC. In the group with dextrocardia, the frequencies of CHD were as follows: RV outlet obstruction (41/65, 63.1%), AVSD (40/65, 61.5%), DORV (37/65, 56.9%), abnormal superior vena cava (36/65, 55.4%), APVC (35/65, 53.8%) and arrhythmia (17/65, 26.1%). RV outlet obstruction included 25 cases with pulmonary valve stenosis and 16 cases with pulmonary artery atresia. Abnormal superior vena cava included 26 cases with double SVC and 10 cases with persistent left SVC. APVC included 30 cases with total anomalous pulmonary venous connection and 5 cases with partial anomalous pulmonary venous connection. Arrhythmia included 10 cases with complete AV block and 7 cases with supraventricular tachycardia.

**Fig. 3:**
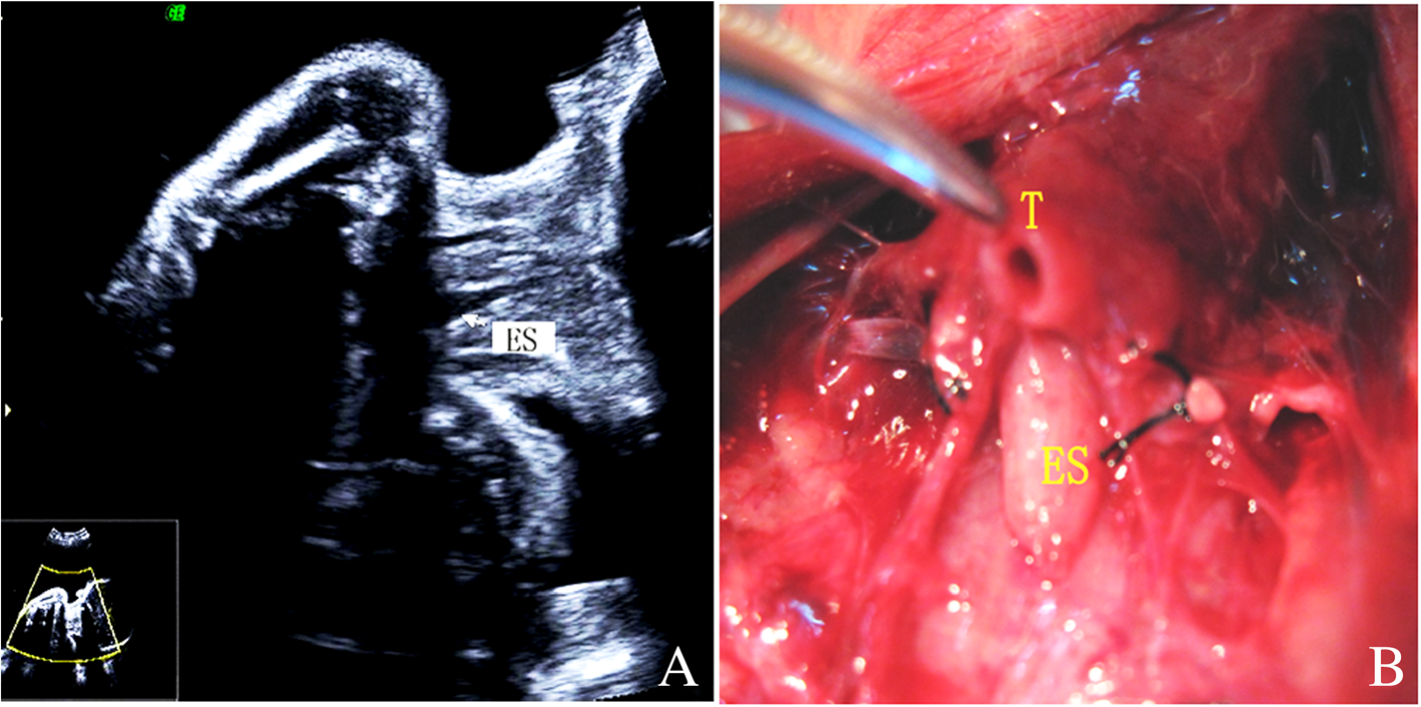
A 22.3-week gestational age fetus with levocardia and esophageal atresia. A cystic dilated esophagus was observed during fetal ultrasound screening (**a**), and autopsy confirmed esophageal atresia (**b**)

**Fig. 4:**
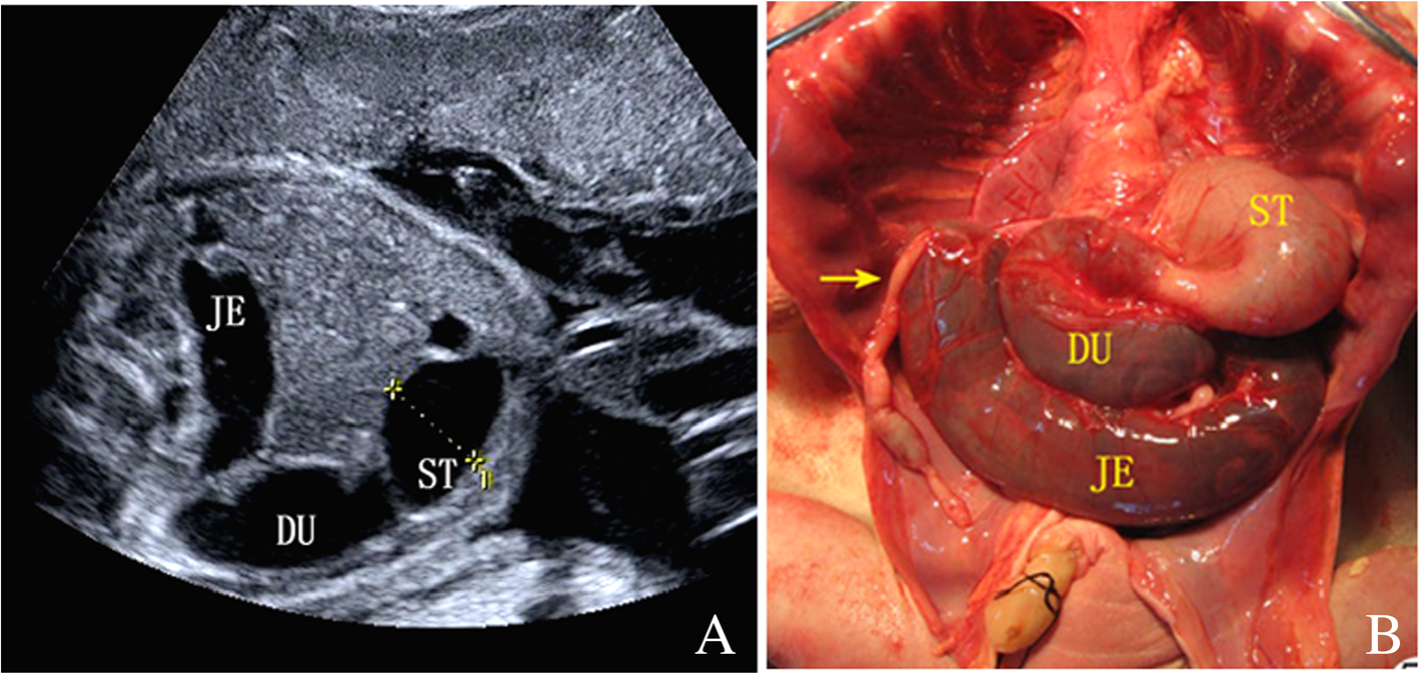
A 24-week gestational age fetus with dextrocardia and intestinal atresia. A dilated jejunum and duodenum were seen during fetal ultrasound screening (**a**), and autopsy confirmed intestinal atresia (**b**)

Few extracardiac deformations were detected prenatally in the enrolled fetuses. There were no differences in the presence of extracardiac and chromosomal anomalies between the two groups (*P* > 0.05). In the group with levocardia, 1 fetus exhibited esophageal atresia (Fig. 3) and a single umbilical artery, 1 fetus had a polycystic kidney, and one fetus had a single UA. In the group with dextrocardia, 1 fetus showed duodenal atresia and a single UA, 2 fetuses had a short long bone, 1 fetus exhibited intestinal atresia (Fig. 4), 1 fetus had a hernia and a single UA, and 1 fetus had unilaterally absent kidneys.

Chromosomal analysis was available in 89 cases (58 with dextrocardia and 31 with levocardia). Chromosomal anomalies were detected in 4 fetuses with levocardia, including one with trisomy 21, one with trisomy18, one with 45X and one with triploidy. Chromosomal abnormalities were detected in 5 fetuses with dextrocardia, including one with trisomy 21, one with 45X, one trisomy 3p, one partial monosomy 7p and one 5p-.

There were no differences in perinatal outcome between the two groups. Approximately half of the enrolled fetuses were live births: 20 fetuses with levocardia (52.7%) and 29 fetuses with dextrocardia (44.6%).

Table 2 shows the postnatal findings and outcomes for the enrolled patients. Most of the cardiovascular lesions were correctly diagnosed in utero, including AVSD, ventricular arterial connections, outlet obstruction, and pulmonary and systemic venous connections (Table 3). However, postnatal ECG demonstrated that many more live infants with dextrocardia had an associated arrhythmia (*P* < 0.05). In the levocardia group, there were 3 infants with a complete AV block, 2 with atrial flutter, 2 with first degree AV block, 1 with sick sinus syndrome and 1 with ventricular tachycardia, whereas in the dextrocardia group, there were 8 infants with complete AV block, 6 with supraventricular tachycardia, 3 with atrial flutter, 3 with second degree AV block, 2 with sick sinus syndrome and 2 with atrioventricular nodal reentry tachycardia.

**Table 2:** Postnatal findings and outcomes of fetuses with levocardia and dextrocardia

Characteristics		Fetus with levocardia (n = 38)	Fetus with dextrocardia (n = 65)	*P*
Postnatal findings				
Isomerism	Left, n (%)	19 (50%)	35 (53.8%)	0.83
	Right, n (%)	14 (40%)	20 (30.8%)	0.66
Cardiovascular issue				
Without defect, n (%)		1 (2.6%)	0	0.37
Any defect, n (%)		37 (97.4%)	65 (100%)	0.37
Arrhythmias, n (%)		9/20 (45%)	24/29 (82.7%)	*P* < 0.05
Extracardiac issue				
Without malformation, n (%)		2 (5.3%)	15 (23.1%)	*P* < 0.05
Any malformation, n (%)		36 (94.7%)	50 (76.9%)	*P* < 0.05
Esophageal atresia, n (%)		3 (7.9%)	0	*P* < 0.05
Duodenal atresia, n (%)		0	2 (3.1%)	0.53
Intestinal atresia, n (%)		0	1 (1.5%)	1.0
Hernia, n (%)		1 (2.6%)	2 (3.1%)	0.37
Renal lesion, n (%)		1 (2.6%)	1 (1.5%)	1.0
Bone malformation, n (%)		0	2 (3.1%)	0.53
Intestinal malrotation, n (%)		33 (86.8%)	45 (69.2%)	0.06
Biliary atresia, n (%)		0	3 (4.6%)	0.67
Brain malformation, n (%)		0	9 (13.8%)	*P* < 0.05
Outcome				
Follow-up period, month		78.1 ± 55.8	69.1 ± 54.3	0.58
Living, n (%)		17/20	18/29	0.11
Death in hospital, n (%)		2/20	7/29	0.28
Death post-discharge, n (%)		1/20	4/29	0.64
Surgery, n (%)		17/20	26/29	0.68

**Table 3:** Accuracy of isomerism classification and intracardiac and extracardiac anomalies during fetal diagnosis

Characteristics		Fetus with levocardia (n = 38)	Fetus with dextrocardia (n = 65)
Isomerism	Left Right	8/19 (42.1%)	11/35 (31.4%)
		7/14 (50%)	9/20 (45%)
Cardiovascular anomalies			
Interrupted IVC		7/7 (100%)	18/18 (100%)
Abnormal SVC		9/9 (100%)	36/37 (97.3%)
APVC		9/11 (81.8%)	35/38 (92.1%)
AVSD		20/20 (100%)	40/40 (100%)
Common atrium		15/15 (100%)	24/24 (100%)
Univentricular physiology		25/25 (100%)	16/16 (100%)
TGA		14/14 (100%)	4/4 (100%)
DORV		4/4 (100%)	37/37 (100%)
DOLV		0/0 (100%)	1/1 (100%)
Truncus arteriosus		2/2 (100%)	0/0
RV outlet obstruction		25/26 (96.2%)	41/41 (100%)
LV outlet obstruction		1/1 (100%)	11/12 (91.7%)
Aortic arch anomaly		6/6 (100%)	6/7 (85.7%)
Extracardiac anomalies			
Asplenia		5/18 (27.8%)	5/18 (27.8%)
Polysplenia		3/12 (25%)	6/33 (18.1%)
Esophageal atresia		1/3 (33.3%)	0/0
Duodenal atresia		0/0	1/2 (50%)
Hernia		0/1	2/2 (100%)
Renal lesion		1/1 (100%)	1/1 (100%)
Bone malformation		0/0	2/2 (100%)
Intestinal malrotation,		0/33 (0%)	0/45 (0%)
Biliary atresia		0/0	0/3 (0%)
Brain malformation		0/0	0/9 (0%)

*APVC* abnormal pulmonary venous connection, *AVSD* atrioventricular septal defect, *DORV* double outlet of right ventricle, *DOLV* double outlet of left ventricle, *IVC* inferior vena cava, *SVC* superior vena cava, *TGA* transposition of the great arteries

Extracardiac abnormalities were detected postnatally in almost of all levocardia cases, including 33 cases with intestinal malrotation (86.8%), 3 cases with esophageal atresia (7.9%), 1 case with hernia and one with polycystic kidney (2.6%). Fewer infants in the dextrocardia group exhibited extracardiac malformations compared with the levocardia group (*P* < 0.05). In the group with dextrocardia, there were 45 cases with intestinal malrotation (69.2%), 9 cases with brain abnormalities (13.8%), 2 cases with duodenal atresia (3.1%), 2 with hernia (3.1%) and one with unilateral absent kidney (1.5%).

Finally, there were 35 living patients at the end of study, including 17 patients with levocardia and 18 with dextrocardia. There were no significant differences in the survival rate, death rate or surgery rate between the two groups (*P* > 0.05).

The accurate diagnosis rates for isomerism classification and spleen malformation during the fetal period were very low (less than 50%). The overall accuracy rate of diagnosing cardiovascular anomalies was high; however, 5 cases of APVC, 1 case of LV outlet obstruction, and 1 case of arch coarctation were missed prenatally. The overall accuracy rate of extracardiac anomalies was very low, especially for intestinal malrotation, biliary atresia, brain malformation, and esophageal atresia (Table 3). Notably, none of the CNS abnormalities were detected prenatally, however there were 9 cases with brain abnormalities were detected postnatally, including 5 with periventricular leukomalacia, 3 with cerebellar agenesis and 1 with Dandy–Walker malformation.

## Discussion

To the best of our knowledge, this study is the first to describe differences in cardiovascular disease, extracardiac anomalies and outcomes between fetuses with levocardia and fetuses with dextrocardia.

Heterotaxy syndrome is classified as right isomerism if the patient exhibits bilateral structures with morphologically right features, such as bilateral trilobed lungs and bilateral morphologic right bronchi, and as left isomerism if the patient presents with morphologically left bilateral features, such as bilateral bilobed lungs and bilateral morphologic left bronchi [9]. Unfortunately, these structural characteristics cannot be detected prenatally. Several associated sonographic signs have been applied to classify isomerisms during the fetal period [3]. Right isomerism is suspected when the fetus presents with situs ambiguous, cardiac defects or juxtaposition of the aorta and IVC. Left isomerism is suspected when the fetus presents with situs ambiguous and an interrupted IVC or heart block. However, prenatal isomerism diagnoses are not always correct; the accuracy of isomerism classification during fetal diagnosis was lower than 50% in our study. Although certain associated anatomic features are typical for each group, there is some overlap between the subgroups. Therefore, in our study, we subdivided fetuses with heterotaxy into “levocardia” and “dextrocardia” groups according to the position of heart, which is easily detected precisely in utero, with the aim of providing useful information for prenatal consultation.

Our study demonstrated that there was a difference in the type of CHD between fetuses with levocardia and fetuses with dextrocardia, although both groups were associated with a wide spectrum of CHD. Compared with the dextrocardia group, more fetuses in the levocardia group exhibited univentricular physiology and TGA. By contrast, more fetuses in the dextrocardia group had APVC, bilateral SVC, DORV and LV outlet obstruction compared with the levocardia group. All six fetuses with levocardia and aortic arch deformation showed involvement of the right aortic arch, whereas of the 6 fetuses with dextrocardia and aortic arch deformation, 3 cases involved an interrupted aortic arch and 3 cases involved aortic arch hypoplasia. Bohun CM [10] also demonstrated that regarding isomerism, 100% of fetuses with dextrocardia showed cardiac malformations, and the predominant subtypes were anomalous systemic and pulmonary veins, inlet valve abnormalities and DORV. No systematic studies of levocardia with regard to heterotaxy syndrome have been performed other than a few case reports. Our study demonstrated that 94.7% of fetuses with levocardia had cardiovascular deformations. Univentricular physiology, RV outlet obstruction, AVSD and TGA predominated in the levocardia group. Interestingly, many living infants with heterotaxy syndrome exhibited arrhythmia, many more than were detected prenatally [33/49 (67.3%) vs. 21/103 (20.3%), *P* < 0.01]. Moreover, arrhythmia predominated in the group with dextrocardia. Infants with dextrocardia were more likely to have supraventricular tachycardia and atrioventricular block, whereas infants with levocardia were more likely to exhibit atrioventricular block.

Our study demonstrated that 83.5% (86/103) of cases with heterotaxy syndrome had extracardiac deformations. Intestinal malrotation was the dominant extracardiac lesion, occurring in 86.8% of fetuses with levocardia and 69.2% of fetuses with dextrocardia. Previous studies have reported that the incidence of intestinal rotational abnormalities in patients with heterotaxy varied from 71% (12/17) [11] to 83% (24/29) [12]. Although most infants with intestinal malrotation were asymptomatic, 12.2% (5/41) developed acute midgut volvulus requiring emergency laparotomy during the follow-up period of our study, including 3 cases with levocardia and 2 cases with dextrocardia. A recent meta-analysis review [13] demonstrated that the incidences of intestinal malrotation and acute midgut volvulus were significantly greater in heterotaxy patients than in the normal population. Therefore, it is important to perform routine screening and monitoring for intestinal rotational abnormalities in the setting of heterotaxy syndrome.

Infants with dextrocardia in our study were more likely to exhibit central nervous system abnormalities during MRI screening; moreover, periventricular leukomalacia and cerebellar agenesis were the dominant lesions. The underlying mechanism is unknown. It might be related to the particular subtype cardiac defects. Among the 9 cases with brain deformation in the dextrocardia group, there were 6 cases with LV outlet obstruction, 2 cases with arch hypoplasia and 1 case with TGA. Previous studies by our group and other authors also demonstrated abnormal brain structures and impaired brain development in fetuses with complex CHD [14–17], particularly cases with left heart diseases and TGA.

Our study has several limitations. First, we did not screen enrolled fetuses using fetal MRI, because MRI was not widely available in our region. If fetal MRI were used, more cases and a higher accuracy of extracardiac malformations would be detected prenatally, benefiting these infants with prompt treatment after birth. Second, the follow-up period was not sufficiently long to evaluate the long-term outcomes and discrepancies between the two groups.

In summary, heterotaxy syndrome is a complex and heterogeneous disorder with a high morbidity and mortality. Although there was a large overlap in terms of cardiac/extra cardiac anomalies, there are several differences between fetuses with levocardia and fetuses with dextrocardia: univentricular physiology, TGA, esophageal atresia are more frequent in fetuses with levocardia, whereas APVC, DORV, LV outlet obstruction, arrhythmias, brain malformation are more frequent in fetuses with dextrocardia. This new classification according to the fetal heart position could provide useful information on detailed follow-up and benefit prenatal counseling and perinatal management. The assessment of cardiac malformations has a high accuracy, although low sensitivity for assessing extracardiac abnormalities, especially intestinal malrotation, esophageal atresia and brain abnormalities, was observed. These malformations are common and might affect outcome. Therefore, thorough and complete postnatal multisystem screening is recommended for fetuses with heterotaxy.

## Abbreviations

APVC: Abnormal pulmonary venous connection
AVSD: Atrioventricular septal defect
CHD: Congenital heart defects
DORV: Double outlet of right ventricle
IVC: Inferior vena cava
LV: Left ventricle
RV: Right ventricle
SVC: Superior vena cava
TGA: Transposition of the great arteries
UA: Umbilical artery
